# Prevalence of smartphone addiction and its associated factors among pre-clinical medical and dental students in a public university in Malaysia

**DOI:** 10.51866/oa.75

**Published:** 2022-09-11

**Authors:** Abdul Hadi Said, Farah Natashah Mohd, Muhammad Zubir Yusof, Nur Afiqah Nadiah Mohd Win, Aisha Najwa Mazlan, Alya Syahira Shaharudin

**Affiliations:** 1 BDS (UM), Adv Dip (Sedation and Special Care Dentistry,(SSCD), MSc in Clinical SSCD, Department of Dental and Maxillofacial, Kulliyyah of Dentistry, IIUM Kuantan Campus, Kuantan, Malaysia. Email: fasha@iium.edu.my; 2 MD (USM), MMed (Fam Med) (UM), Department of Family Medicine, Kulliyyah of Medicine, IIUM Kuantan Campus, Kuantan, Malaysia.; 3 Ph.D Occupational Hygiene (Aberdeen University), Department of Community Medicine, Kulliyyah of Medicine, IIUM Kuantan campus, Kuantan, Malaysia.; 4 MBBS (IIUM), Hospital Tuanku Ampuan Najihah, Jalan Melang, Kampung Gemelang, Kuala Pilah, Negeri Sembilan, Malaysia.; 5 MBBS(IIUM), Hospital Kemaman, Jalan Da' Omar, Chukai, Terengganu, Malaysia.; 6 MBBS(IIUM), Hospital Kemaman, Jalan Da' Omar, Chukai, Terengganu, Malaysia.

**Keywords:** Smartphone addiction, Pre-clinical, Medicine and dental students

## Abstract

**Introduction:**

Smartphone addiction is becoming a global concern affecting every part of society, including healthcare professionals. This study aimed to identify the prevalence of risk of smartphone addiction and its associated factors among medical and dental students in a public university in Malaysia.

**Method:**

This cross-sectional study was conducted among pre-clinical medical and dental students using convenience sampling. Questions regarding sociodemographic profile and responses to the Smartphone Addiction Scale Short Version (SAS-SV) and Depression, Anxiety and Stress Score questionnaire (DASS-21) were collected. Multiple logistic regression testing was used to analyse the factors associated with smartphone addiction.

**Results:**

We invited 409 pre-clinical medical and dental students to participate voluntarily, resulting in a response rate of 80.2%. The prevalence of high-risk smartphone addiction among the participants was 47.9%. Male participants, participants who used smartphones mainly for social media, and participants with depressive symptoms were more likely to have a high risk of smartphone addiction. Medical students, participants who spent less than 3 hours per day on a smartphone, and participants who used smartphones for education-related activities were less likely to have a high risk of smartphone addiction.

**Conclusion:**

Smartphone addiction prevalence among pre-clinical medical and dental students was high. Therefore, the authorities should overcome this problem by implementing early measures.

## Introduction

The revolution of smartphones brought about numerous benefits, especially with the emergence of the internet. In addition to phone calls and text messages, many other useful applications could be easily installed on smartphones.^1^ Smartphones are extensively used in the delivery of healthcare services, including record-keeping, medical references, and billing. Furthermore, they help improve communication between hospital medical staff and enhance telemedicine capability.^2^ From medical and dental students' perspectives, smartphones allow them to access information easily and quickly.

Despite all the benefits and conveniences that they offer, there is a growing concern regarding the potential negative effects of excessive smartphones use on the psychology and behaviour of the individual, namely addiction. Addiction can be defined as a phenomenon that manifests with tolerance, withdrawal symptoms, and dependence, and is accompanied by social problems.^3^ Previous studies have defined smartphone addiction using various terms. One of the most common definitions for smartphone addiction refers to ‘dependency, excessive and uncontrolled use of smartphone’.^4^ ‘Smartphone addiction’ can be considered one form of technological addiction.^5^ Griffiths (1996) operationally defined technological addictions as a behavioural addiction involving human-machine interaction that is non-chemical.^6^

Smartphone addiction has numerous harmful effects, as it alters quality of life, including efficacy, productivity, sleep patterns, physical activity, and the behaviour of the affected individual.^1,3,5,7^'^8^ Even though smartphones improve the communication efficacy of health providers, smartphone addiction was found to reduce self-efficacy and productivity among those affected.^9^'^10^ Smartphone addiction also can lead to poor sleep quality and reduced physical activity,^1,3,5,7^ and it can harm other people if negligence or ignorance occurs in public, such as using a smartphone while driving, which may cause automobile accidents.^3^

Previous studies have reported a high prevalence of smartphone addiction among medical and dental students, both in local and international settings.^7,11^'^13^ This illustrates that most students are becoming too dependent on phones. The primary concern for educators is that usage of a smart device might negatively impact students’ academic performance. In addition, Malaysia is facing increasing psychological problems, such as depression, anxiety, and stress.^14^'^15^ Interestingly, researchers have reported that these mental health problems are significantly associated with smartphone addiction.^5,7,16^'^17^ The findings are consistent in studies in which smartphone addiction is associated with poor mental health status among university students.^5,7^'^8,16^'^17^ However, there is a paucity of similar local studies, especially among undergraduate medical and dental students; therefore, the objective of our study was to assess this association in a more relevant setting.

The aim of this study is to measure the prevalence of smartphone addiction in pre' clinical medical and dental students at the International Islamic University Malaysia (IIUM) Kuantan Campus. Furthermore, this study aims to study the possible factors associated with smartphone addiction. Identifying these factors will aid in early detection and management of this problem. Factors found to be related to smartphone addiction from previous studies include age, gender, financial status, physical activity, sleep problems, and mental health status.^1,3,5,7,8^ Our study includes several other factors: the students’ faculty (medical or dental), parental marriage status, family history of psychiatric illness, smoking, and scholarship status. We were interested in whether these factors had a significant association with smartphone addiction. By identifying the factors contributing to smartphone addiction, the authorities may take proper measures by creating awareness and planning for early intervention to curb this major problem.

## Methods

### Study design, population and sample size

A cross-sectional study was conducted at IIUM Kuantan Campus among year 1 and year 2 medical and dental pre-clinical students from 15 th October 2018 to 25 th August 2019 using convenience sampling. This study focused on pre-clinical students as they are considered newcomers to the university, and some are new owners of smartphones. We were interested in understanding how well the students coped with different environments, far from parental guidance, in terms of smartphone use and mental health problems. The prevalence of smartphone addiction among local university students in a previous study was 47.7%.^8^ Using the single-proportion formula, the minimum required sample size was 250, with a precision of 0. 05 and considering 20% non-response rate.

### Study instruments and data collection

A validated Smartphone Addiction Scale Short Version (SAS-SV) was used to screen the prevalence of smartphone addiction among the students in healthcare faculties.^3^ The scale consists of 10 questions. Each question is given a score: 1=strongly disagree, 2=disagree, 3=weakly disagree, 4=weakly agree, 5=agree, or 6=strongly agree. Its reliability was shown to be excellent, with a Cronbach’s alpha value of 0.911. The cut-off value for high risk of smartphone addiction was >31 and >33 for males and females, respectively.

All data were collected using a self-administered questionnaire, which was divided into three parts. Part A included information on the participant’s sociodemographic characteristics, which included faculty,gender, race, marital status, scholarship status, household income, regular exercise, sleep problems, smoking, parental status, family history of psychiatric problems, number of smartphones owned, time spent on a smartphone, and main usage of the smartphone. The reason for usage of smartphones was divided into 1, ‘browsing the internet not related to education activities’, which referred to any web browsing ot related to medical or dental education; 2, ‘social media’, which referred to browsingany social media application, such as ‘Facebook’, ‘Instagram’, or ‘Twitter’; 3, ‘basic phone services’, which referred to basic services, such as ‘making a call’ and ‘texting’; 4, games; and 5, ‘education-related activities’, which referred to any activities using smartphones for education purposes, including website browsingand video calls for teaching purposes.

Part B screened for the presence of smartphone addiction among the students of healthcare faculties using the validated Smartphone Addiction Scale Short Version (SAS-SV).^3^ Based on a previous local study, we used 3 hours per day as the cut-off point to identify participants with a smartphone addiction.^12^ Part C determined the presence of depression, anxiety, and stress among the participants using the validated Depression, Anxiety and Stress Scale 21 (DASS-21).^18^ The conditions were further classified as normal, mild, moderate, severe, or extremely severe. Only participants with normal levels in these three components were grouped as having no depression, anxiety, or stress. The DASS-21 is one of the most used questionnaires to assess mental health problems and was validated several years earlier. This questionnaire also possesses excellent psychometric properties, with a Cronbach's alpha of 0.81 for depression, 0.89 for anxiety, and 0.78 for stress subscales.^19^

### Data analysis

Data entry and analysis were performed using IBM SPSS version 25.0. Categorical variables are reported as frequencies and percentages. Sociodemographic characteristics and level of depression, anxiety, and stress were analysed using a chi-square test and Fisher’s exact test. A 95% confidence interval (CI) was used, and a p-value <0.05 was considered statistically significant. Later, the factors associated with smartphone addiction were analysed using a binary logistic regression test. The odds ratio (OR) at 95% CI was reported to measure the likelihood of the factors being associated with smartphone addiction.

### Ethical considerations

This study was approved by the Kulliyyah of Medicine and IIUM Research Ethics Committee (IREC), ID no. IREC 2019143. Participation was entirely voluntary. A written consent form was signed by all participants who agreed to participate in the survey. Furthermore, they were well-informed that their data confidentiality was preserved. Participants who were found to have severe or extremely severe anxiety, stress, or depression were contacted and advised to seek treatment at our clinic.

## Results

Three hundred and twenty-eight students participated in this study; 225 students were from Faculty of Medicine and 102 students were from Kulliyyah of Dentistry, with a response rate of 80.2%.

The participants' sociodemographic profiles and mental health statuses are summarised in **Table 1**. Most participants owned only one smartphone (88.4%) and spent more than 3 hours per day on their smartphone (79.9%). The three main reasons for smartphone usage among the participants were social media (78.0%), browsing the internet not related to educational activities (53.4%), and education-related activities (43.9%).

Regarding mental health status, 41.2% of the participants had symptoms of depression, but most had only mild-to-moderate symptoms. More than half of the participants had symptoms of moderate-to-extremely severe anxiety (50.2%), but their stress levels were mostly normal (66.2%).

**Table 1:** Sociodemographic profile and mental health status of the participants.

Variable	n	%
Medicine	225	68.6
Dentistry	103	31.4
*Gender*		
Female	246	75.0
Male	82	25.0
*Race*		
Malay	322	98.2
Non-Malay	6	1.8
*Marital status*		
Single	328	100.0
Married	0	0.0
*Scholarship status*		
Yes	159	48.5
No	169	51.5
*Household income*		
B40	83	26.3
M40	89	28.2
T20	144	45.6
*Regular exercise*		
Yes	117	35.7
No	211	64.3
*Sleep problems*		
Yes	103	31.4
No	225	68.6
*Parents’ marital status*		
Married	305	93.0
Divorce	23	7.0
*Smoking status*		
Smoker	5	1.5
Non-smoker	323	98.5
*Family history of psychiatric problems*		
Yes	18	5.5
No	310	94.5
*Number of smartphones owned*		
≤1	290	88.4
≥2	38	11.6
*Time spent on a smartphone (hours/day)*		
>3	262	79.9
0-3	66	20.1
*Smoking status*		
Smoker	5	1.5
Non-smoker	323	98.5
**Main usage of smartphone**		
*Surfing the internet not related to educational activities*		
Yes	175	53.4
No	153	46.6
*Social media*		
Yes	256	78.0
No	72	22.0
*Basic phone services*		
Yes	130	39.6
No	198	60.4
*Games*		
Yes	61	18.6
No	267	81.4
*Education-related activities*		
Yes	144	43.9
No	184	56.1
**Level of depression, anxiety, and stress**		
*Depression*		
Normal	193	58.8
Mild	43	13.1
Moderate	54	16.5
Severe	20	6.1
Extremely severe	18	5.5
*Anxiety*		
Normal	126	38.5
Mild	37	11.3
Moderate	63	19.3
Severe	45	13.8
Extremely severe	56	17.1
**Level of depression, anxiety, and stress**		
*Stress*		
Normal	217	66.2
Mild	49	14.9
Moderate	35	10.7
Severe	20	6.1
Extremely severe	7	2.1

**Figure 1:**
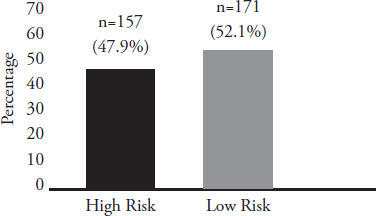
Prevalence of the risk of smartphone addiction among the participants.

The factors associated with smartphone addiction are presented in **Table 2**. There was a significant association between the risk of smartphone addiction and Kulliyyah, gender, sleep problems, time spent on the smartphone, usage of smartphones for social media and for education-related activities. The result revealed a significant relationship between risk of smartphone addiction and depression (p=0.001) and stress (p=0.007).

**Table 2:** Association between sociodemographic profile, mental health status, and risk of smartphone addiction.

Variable	Risk of smartphone addiction		χ^2^	P
	High risk n (%)	Low risk n (%)		
*Kulliyyah*				
Medicine	98 (62.4)	127 (74.3)	5.33	0.021^*^
Dentistry	59 (37.6)	44 (25.7)		
*Gender*				
Female	104 (66.2)	142 (83.0)	12.32	<0.001^*^
Male	53 (33.8)	29 (17.0)		
*Race*				
Malay	155 (98.7)	167 (97.7)	0.52	0.472
Non-Malay	2 (1.3)	4 (2.3)		
*Scholarship status*				
Yes	70 (44.6)	89 (52.0)	1.82	0.177
No	87 (55.4)	82 (48.0)		
*Household income*				
B40	41 (26.5)	42 (26.1)	0.44	0.801
M40	46 (29.7)	43 (26.7)		
T20	68 (43.9)	76 (47.2)		
*Regular exercise*				
Yes	51 (32.5)	66 (38.6)	1.33	0.248
No	106 (67.5)	105 (61.4)		
*Sleep problems*				
Yes	60 (38.2)	43 (25.1)	6.50	0.011^*^
No	97 (61.8)	128 (74.9)		
*Parents' marital status*				
Married	146 (93.0)	159 (93.0)	0.00	0.997
Divorce	11 (7.0)	12 (7.0)		
*Smoking status*				
Smoker	4 (2.5)	1 (0.6)	2.10	0.197
Non-smoker	153 (97.5)	170 (99.4)		
*Family history of psychiatric problems*				
Yes	12 (7.6)	6 (3.5)	2.70	0.100
No	145 (92.4)	165 (96.5)		
*Number of smartphones owned*				
≤1	138 (87.9)	152 (88.9)	0.08	0.779
≥2	19(12.1)	19(11.1)		
*Time spent on a smartphone (hours/day)*				
>3	138 (87.9)	124 (72.5)	12.05	**<0.001** ^*^
0-3	19 (12.1)	47 (27.5)		
*Main usage of smartphone*				
*Surfing the internet not related to educational activities*				
Yes	66 (58.0)	84 (49.1)	2.66	0.109
No	66 (42.0)	87 (50.9)		
*Social media*				
Yes	131 (83.4)	125 (73.1)	5.11	0.024^*^
No	26 (16.6)	46 (26.9)		
*Basic phone services*				
Yes	60 (38.2)	70 (40.9)	0.25	0.615
No	97(61.8)	101 (59.1)		
*Games*				
Yes	31 (19.7)	30(17.5)	0.26	**0.609**
No	126 (80.3)	141 (82.5)		
*Education-related activities*				
Yes	56 (35.7)	88 (51.5)	8.29	**0.004** ^*^
No	101 (64.3)	83 (48.5)		
**Level of depression, anxiety, and stress**				
*Depression*				
Yes	80 (51.0)	55 (32.2)	11.94	**0.001** ^*^
No	77 (49.0)	116(67.8)		
*Anxiety*				
Yes	103(65.6)	98 (57.6)	2.18	0.172
No	54 (34.4)	72 (42.4)		
*Stress*				
Yes	65 (41.4)	56 (26.9)	7.69	0.007^*^
No	92 (58.6)	125 (73.1)		

* p-value <0.05

Multiple logistic regression analysis was performed to evaluate the factors associated with risk of smartphone addiction (**Table 3**). Male participants, participants who used the smartphone mainly for social media, and participants with depression were more likely to have high risk of smartphone addiction. Participants from Kulliyyah of medicine, participants who spent less than 3 hours per day on their smartphone, and participants who used their smartphones for education-related activities were less likely to have a high risk of smartphone addiction. Sleep problems and having anxiety and stress symptoms were not significantly associated with smartphone addiction.

**Table 3:** Multiple logistic regression analysis to determine factors associated with high risk of smartphone addiction.

Variable	B	Wald	AOR+	95% CI^#^	P
*Kulliyyah*					
Dentistry (reference)					
Medicine	0.57	4.68	0.56	0.34-0.95	0.03*
*Gender*					
Female (reference)					
Male	0.80	7.61	2.23	1.26-3.94	0.01*
*Sleep problems*					
Yes (reference)					
No	0.24	0.83	0.79	0.47-1.32	0.36
*Time spent on smartphone (hours per day)*					
>3 (reference)					
0-3	0.87	7.40	0.42	0.23-0.79	0.01*
*Use of smartphone for social media*					
No (reference)					
Yes	0.63	4.08	1.87	1.02-3.43	0.04*
*Use of smartphone for education-related activities*					
No (reference)					
Yes	0.70	7.24	0.50	0.30-0.83	0.01*
*Depression*					
No (reference)					
Yes	0.63	5.85	1.87	1.13-3.12	0.02*
*Stress*					
No (reference)					
Yes	0.34	1.60	1.41	0.83-2.39	0.21

## Discussion

The prevalence of participants with high-risk smartphone addiction was 47.9%, which was marginally lower than a local study conducted among medical students at UiTM Sungai Buloh and Selayang Campus, where the prevalence was more than one-half.^12^ In contrast, another local study conducted at a private university reported that only one-third of dental students exhibited excessive smartphone use.^13^ This difference might be due to different tools used to measure the phenomenon. The dental students were interviewed using a pre-tested and selfrated questionnaire, resulting in a subjective estimation of the problem. In addition, there was a notable difference in the prevalence of smartphone addiction compared to international findings. A study conducted in China found a lower prevalence of smartphone addiction (29%).^7^ In contrast, a study conducted in Saudi Arabia reported a higher prevalence of smartphone addiction (71%).^20^ Therefore, the prevalence of smartphone addiction among medical and dental students varies between countries, and it would be of interest to study the reasons for these differences.

This study further investigated the pattern of smartphone use: most participants spent more than 3 hours per day on their smartphone, and their primary use was social media, which was consistent with a local study among medical students and staff at Malaysian public universities.^12^ Moreover, a study conducted in Saudi Arabia reported that most dental students spent more than 3 hours on their smartphones; however, most of these students used their smartphones for web surfing.^20^ Therefore, smartphone usage is part of the daily activities for most medical and dental students. Smartphone addiction is a major problem in this era both globally and locally. Because smartphone addiction can lead to poor productivity and quality of life among students, which will affect their future careers, the authorities should take proper measures regarding mental health problems related to this technology by focusing more research on factors associated with smartphone addiction, creating awareness, and planning early interventions to minimise this problem.

Dental students were found to be more likely than medical students to have a high risk of smartphone addiction. This finding was likely due to pre-clinical-year dental students having a more open schedule than pre-clinical-year medical students, allowing them more time to spend on their smartphones. Surprisingly, there have been no studies comparing the risk of smartphone addiction in students in medical and dentistry. In our study, males had a significantly higher risk of smartphone addiction than females. This finding was inconsistent with a study conducted among medical and dental students in Malaysia and China that showed no significant variation among genders.^7,12^ However, a study conducted among university students in other programmes reported that females had a higher prevalence of smartphone addiction than males.^21^ Due to inconsistencies in findings regarding the prevalence of smartphone addiction among males and females, further studies with a larger sample size are necessary to clarify the inconsistent results.

Our study found that smartphone use for social media and educational purposes were significant determinants smartphone addiction; participants who used smartphones primarily for social media had a higher risk of smartphone addiction, and vice versa for those who used smartphones primarily for educational purposes. Previous research showed that social networking was one of the significant predictors of smartphone addiction among university students in the United States.^22^ This could be because the population studies involved young adults, an age group in which social networking is the preferred medium for socialising via smartphones. Otherwise, no research has been published on the relationship between smartphone use for education and the risk of smartphone addiction. Fortunately, this discovery in our study suggests that smartphones used for educational purposes can be promoted to students without fear of smartphone addiction.

Furthermore, participants with depression were more likely to have a high risk of smartphone addiction than participants without depression. This finding was supported by a previous study that identified depression as one of the major initiators of internet addiction.^23^ The current study's findings are consistent with a study conducted among medical students in China that revealed a significant relationship between smartphone addiction and depression.^7^ The possible reason for this association is that students who have depression might use their smartphones as an escape from feeling depressed. A previous study found that smartphone use could act as an avoidance strategy to aversive emotional content.^7^ In addition, people with depression may have sleep problems and could use a smartphone throughout the night in attempt to overcome this problem; this could occur repeatedly, which may lead to smartphone addiction. It would be interesting to determine whether the use of smartphones can help people with depression improve their symptoms or if it makes their symptoms worse.

Unfortunately, this study failed to find a link between sleep problems, anxiety, stress symptoms, and smartphone addiction. It differed from studies conducted in China and Lebanon, where these variables were discovered to be significant independent factors for smartphone addiction.^7,16,25^ These findings may indicate that medical and dental students in the our study did not use smartphones as a coping mechanism against sleep or psychological problems, including anxiety and stress. Therefore, it would be of interest for future research to identify our students’ coping mechanisms.

There were several limitations of this study. First, the prevalence of smartphone addiction in this study cannot be generalised to the general population due to non-random sampling. However, the sample size was large and adequate to represent pre-clinical medical and dental students in IIUM Kuantan Campus, which allowed us to observe statistically significant results. Moreover, the present study had a sufficient response rate as the participants cooperated well in this survey. Second, the study tools used to determine the participants’ sociodemographic profiles depended on self-reported measures that could have been overestimated, such as the questions regarding sleep problems and regular exercise. Otherwise, the tools used to measure the risk of smartphone addiction and level of depression, anxiety, and stress among the participants were validated. Other limitations include recall and reporting bias, as participants had to recall their time spent on the smartphone and may have omitted some information, such as smoking status and monthly household income. Nonetheless, the current study had reliable responses, as it used fixed-response questions, which reduced the variability in the results that differences among the interviewers may have caused.

Some recommendations for future research can be implemented. To accurately generalise the results to the population, researchers should use probability sampling techniques, such as random sampling. Furthermore, a future study population could be expanded to a larger geographical area involving multiple universities from both public and private sectors. For more accurate statistical analysis, we recommend that each group for each independent variable have an equal number of participants. Furthermore, it is recommended that appropriate agencies conduct more research on emerging mental health problems associated with rapidly evolving technologies.

## Conclusion

The risk of smartphone addiction among pre-clinical medical and dental students at IIUM Kuantan was high. Of all the variables studied, male gender, depression, and using a smartphone primarily for social media were associated with a high risk of smartphone addiction. Medical students, who spent less than 3 hours per day on a smartphone and used smartphones mainly for education-related activities were less likely to have a smartphone addiction. Because it is well known that smartphone addiction can have negative impacts, it is essential to raise awareness of the high prevalence of smartphone addiction and to plan for early intervention among these future doctors and dentists
